# Nematode control in suckler beef cattle over their first two grazing seasons using a targeted selective treatment approach

**DOI:** 10.1186/s13620-015-0038-1

**Published:** 2015-06-18

**Authors:** James O’Shaughnessy, Bernadette Earley, John F. Mee, Michael L. Doherty, Paul Crosson, Damien Barrett, Theo de Waal

**Affiliations:** Animal and Bioscience Research Department, Animal & Grassland Research and Innovation Centre, Teagasc, Grange, Dunsany, Co. Meath, Ireland; School of Veterinary Medicine, University College Dublin, Belfield, Dublin 4, Ireland; Animal and Bioscience Research Department, Animal & Grassland Research and Innovation Centre, Teagasc, Moorepark, Fermoy, Co. Cork, Ireland; Livestock Systems Department, Animal & Grassland Research and Innovation Centre, Teagasc, Grange, Dunsany, Co. Meath, Ireland; DAFM, Sligo Regional Veterinary Laboratory, Doonally, Co. Sligo, Ireland

**Keywords:** Anthelmintics, Gastrointestinal nematodes, Lungworm, Suckler beef cattle, Targeted selective treatments

## Abstract

**Background:**

With concerns over the development of anthelmintic resistance in cattle nematode populations, we must re-examine our approach to nematode control in cattle. Targeted selective treatments (TST), whereby individual animals are treated instead of entire groups, are being investigated as an alternative. The study objective was to determine if anthelmintic usage could be reduced using a TST-based approach to nematode control in spring-born suckler beef cattle over their first and second grazing seasons (SGS) without affecting performance. In the first grazing season (FGS), 99 calves with an initial mean (s.d.) calf age and live weight on day 0 (June 28^th^ 2012) of 107 (23.1) days and 160 (32.5) kg, respectively, were used. The study commenced on day 0 when calves were randomised and allocated to one of two treatments; 1), standard treatment (control) and 2), TST. Control calves were treated subcutaneously with ivermectin on days 0, 41 and 82 in the FGS. All calves were treated with ivermectin on day 124 and housed on day 133. In the SGS, only heifer calves from the FGS were used and control heifers were treated with ivermectin on day 393. Animals were weighed, blood and faecal sampled every three weeks. The TST animals were treated with ivermectin if thresholds based on a combination of plasma pepsinogen concentrations, faecal egg count and/or the presence of *Dictyocaulus viviparus* larvae in faeces (FGS only) were reached.

**Results:**

No TST calves reached the treatment threshold criteria in the FGS. The FGS average daily live weight gain (ADG ± s.e.m.) for control and TST group calves was 0.89 ± 0.02 kg and 0.94 ± 0.02 kg day^−1^, respectively (*P* = 0.17). In the SGS, all heifers were treated with ivermectin on day 431 due to clinical signs of respiratory disease. The ADG for control and TST heifers from turnout on day 321 to day 431 was 0.90 ± 0.04 and 0.80 ± 0.04 kg day^−1^, respectively (*P* = 0.03).

**Conclusions:**

Spring-born FGS suckler beef calves require minimal anthelmintic treatment to maintain performance. In contrast, clinical parasitic disease may develop in the SGS unless appropriate anthelmintic treatment is provided.

## Background

In contrast to other European countries, cattle in Ireland experience a greater exposure to gastrointestinal nematode (GIN) challenge [1]. This is mainly due to both the greater proportion of grass in the diet and the higher levels of rainfall compared to other European countries [2]. Within such production systems, anthelmintics are administered to ensure that animal health and performance (e.g. live weight gain) are not compromised due to nematode challenge.

At present, there is little published information on the control of infections due to both GIN and *Dictyocaulus viviparus* (lungworm) under Irish environmental conditions for first and second grazing season (SGS) suckler beef cattle. In a previous study, we reported that spring-born suckler beef calves did not require anthelmintic treatment during the first grazing season (FGS) under Irish conditions [3], provided appropriate control measures were taken to prevent dictyocaulosis from occurring. These findings are in agreement with international studies where the risk of parasitic gastroenteritis (PGE) developing in spring-born suckler beef calves in the FGS is regarded as low [4–6]. However, PGE may develop in weaned beef calves [7] in a similar pattern to PGE in FGS dairy calves.

With regards to SGS cattle, a study evaluating the effects of two anthelmintic treatments with topical ivermectin on the performance of suckler beef cattle concluded that the risk of PGE developing during the SGS was governed mainly by animal age, with suckler beef calves born late in the FGS (March - July) developing PGE in their SGS [8]. However, the study did not provide details on the level of parasite exposure or of the treatments provided to calves in their FGS.

Given that the majority of suckler calves in Ireland are born in the spring [9], there is a need to establish guidelines for the control of parasitic challenges due to both GIN and lungworm infection in spring-born suckler beef cattle in their SGS. In addition, there have been an increasing number of reports of anthelmintic resistance in cattle nematode populations worldwide [10–12]. Consequently, sustainable alternatives are being sought for the control of nematode infections in cattle. Targeted selective treatments (TST) are one example of such an alternative that are now being evaluated [13], where control is based on treatment of individuals as opposed to entire groups. This refugia-based approach [14], the aim of which is to reduce our reliance on anthelmintics, has seldom been examined in cattle [15, 16]. Both these TST-based studies of dairy-bred beef cattle reported reductions in anthelmintic use of 65 to 92 %. However, given the differences that exist in the epidemiology of GIN infections between dairy and suckler beef cattle [4, 17], this approach may not be appropriate for suckler beef cattle. We have previously reported on a TST approach in FGS suckler beef calves [3]; however, the effects of this approach conducted over both the FGS and SGS have not been investigated.

The study hypothesis was that using a TST-based approach to nematode control, excluding a pre-housing treatment, spring-born suckler beef calves would require minimal anthelmintic treatments in their FGS and would achieve similar levels of live weight gain to calves receiving three anthelmintic treatments. In their SGS, it was hypothesised that these calves would require at least one anthelmintic treatment to prevent PGE from occurring.

## Methods

All animal procedures were conducted under experimental licence (B100/2869) from the Irish Department of Health and Children in accordance with the Cruelty to Animals Act 1876 and the European Communities (Amendment of Cruelty to Animals Act 1876) Regulation 2002 and 2005.

### Study design

The study was conducted over two grazing seasons on a 70 hectare (ha) farmlet at the Animal & Grassland Research and Innovation Centre, Teagasc, Grange, Dunsany, Co. Meath, Ireland (longitude 6 ° 40' W; latitude 53 ° 30' N; elevation 92 m above sea level).

In the FGS, study animals consisted of 86 Charolais and 13 Blonde d’Aquitaine-sired single-suckled spring-born beef calves and their dams. The breed of dams were as follows; Charolais × Limousin (*n* = 27), Charolais × Simmental (*n* = 26), Limousin × Friesian (*n* = 25) and Limousin × Simmental (*n* = 21). The study commenced on June 28^th^ 2012 (day 0) when calves, which had never been treated with anthelmintics, were randomised based on calf age, live weight, sex, dam breed and sire breed and allocated to one of two treatments; 1), standard treatment (positive control) (*n* = 25; × 2) and 2), TST (*n* = 25; × 1 and *n* = 24; × 1). Mean (s.d.) calf age and live weight on day 0 were 107 (23.1) days and 160 (32.5) kg, respectively. All calves in the positive control groups were treated subcutaneously with ivermectin (1.0 ml per 50 kg bodyweight, Qualimec® 10 mg/ml Solution for Injection, ECO Animal Health Limited) on days 0, 41 and 82. Individual calves in the TST groups were treated at pasture with the same product at the same dosage rate when one of the following thresholds were met; 1), positive for lungworm larvae using the modified Baermann technique or 2), positive or negative for lungworm larvae using the modified Baermann technique with plasma pepsinogen concentrations (PP) ≥ 2 international units of tyrosine/l (Utyr) and faecal egg count (FEC) ≥ 200 eggs per gram of faeces (epg). All calves, in both treatment groups, were treated subcutaneously with ivermectin on day 124 and housed on day 133.

In the SGS, only heifer calves from the FGS were used. Heifers from the FGS (*n* = 46) were maintained in the same treatments during the SGS and were co-grazed (*n* = 12 control and *n* = 11 TST heifers; × 2). Both groups were turned out to pasture in the SGS on day 251 (March 6^th^ 2013). Due to adverse weather conditions which resulted in poor underfoot conditions, heifers of both groups were rehoused on day 253 until turnout again on day 321. All control heifers were treated subcutaneously with ivermectin on day 393. Targeted selective treatment heifers were only eligible for treatment at pasture with the same product as the control heifers if predetermined thresholds were reached [PP ≥ 2.0 Utyr and FEC ≥ 200 epg]. Heifers were housed on day 468 for a 147 day grazing season.

### Sample collection

In the FGS, bull and heifer calves were weighed, blood and faecal sampled approximately every three weeks on days 0, 19, 41, 61, 82, 103 and 124. In the SGS, heifers were weighed, blood and faecal sampled approximately every three weeks on days 251, 279, 300, 321, 342, 363, 384, 405 and 426.

Faecal egg counts were determined using a modified McMaster method with a limit of detection of 50 epg [18] while PP were also measured [19]. In the FGS, faecal samples (10 g per calf) were also analysed for the presence of lungworm larvae using the modified Baermann technique as previously described [20]. Blood samples collected from calves in the FGS on days 0 and 124 and on day 468 at the end of the SGS were tested for the presence of antibodies to lungworm at UCD Veterinary Diagnostic Laboratories with an enzyme-linked immunosorbent assay (ELISA) using recombinant major sperm protein as antigen [21]. Plasma copper values were quantified during both grazing seasons on days 0, 124, 251, 342 and 384 using a Varian Techtron Atomic Absorption spectrophotometer, Model 220, by dilution and direct spray and expressed as μmol/l.

Faecal cultures were performed for each treatment group (2 g of faeces per heifer) in the SGS on days 384, 405 and 426. Cultures were incubated at 27 °C for eight days and 100 L_3_ larvae per culture were identified to genus level using standard identification keys [22]. All L_3_ larvae were identified when counts were less than 100. Faecal samples were analysed for the presence of liver and rumen fluke eggs using a sedimentation technique on day 426 [23].

In the SGS, grass samples were collected every three weeks to determine pasture L_3_ larval burdens using a previously described method [3]. Briefly, one collector was used per plot and grass samples were taken along a double “W” pattern using scissors. A 100 g sub-sample was removed for dry matter estimation.

In the FGS, calf average daily live weight gain (ADG) was calculated as follows: calves were weighed on day 0 and again on day 124. The ADG was calculated as the total live weight gain divided by the number of days (*n* = 124). In the SGS, heifer ADG was similarly calculated except that ADG was calculated from both turnout on day 321 to anthelmintic treatment of all heifers on day 431 and from turnout to housing on day 468, respectively.

### Animal health

Yearling heifers were vaccinated against lungworm infection (Bovilis Huskvac, Intervet Ireland Limited) on days 287 and 315.

### General animal and pasture management

In the FGS, cows with their calves were turned out to pasture in batches after calving from day −107 to day −43 (day −87 mean turnout) and were rotationally grazed together on a predominantly perennial ryegrass-based (*Lolium perenne*) pasture [3].

Calves were weaned on day 110 in one batch and cows were housed on the same day. Weanlings remained at pasture and were allocated into six groups based on age and sex. Concentrates were introduced to weanlings at pasture on day 113 (one kilogram of concentrate head^−1^ day^−1^). All weanlings were housed on day 133. Weanlings were offered 1^st^ cut silage ad-libitum (73 % dry matter digestibility) plus one kilogram of concentrate head^−1^ day^−1^ during the housing period. Weanlings were treated subcutaneously with an ivermectin/closantel combination treatment (1 ml per 25 kg bodyweight, Closamectin Solution for Injection for Cattle and Sheep, Norbrook Laboratories Limited) approximately six weeks post-housing.

In the SGS, heifers were rotationally grazed within the 70 ha farmlet. The heifer grazing block comprised of 15 paddocks, all of which had been predominantly grazed by SGS spring-born suckler beef cattle in the previous grazing season. Mean paddock area was 0.89 ha (range 0.77 to 1.22 ha). Target pre- and post-grazing sward heights were 10 to 12 cm and 4 cm, respectively. Heifers were only moved to other areas of the farmlet when target pre-grazing sward heights were not reached or to graze the headlands of grass silage fields immediately post-harvest.

### Statistical analysis

Normality of data distribution was tested using the PROC UNIVARIATE procedure of SAS 9.3. Data that were not normally distributed (FEC, PP and plasma copper) were transformed by raising the variable to the power of lambda. The required lambda value was calculated by conducting a Box-Cox transformation analysis using the TRANSREG procedure of SAS. Data subjected to transformations were used for P-values. However, the corresponding least squares means (Lsmeans) and standard error of the mean (± s.e.m.) are presented to facilitate interpretation of results. The MIXED procedure of SAS was used to examine the effect of treatment on ADG, FEC, PP and plasma copper. The statistical model used included the fixed effects of treatment, time, gender (FGS), dam breed, sire breed and their interactions. Model effects were considered statistically significant when the type I error rate was less than 5 %. Variables having multiple observations such as FEC, PP and plasma copper were analysed using repeated measures with terms for treatment group, time, gender (FGS), dam breed, sire breed and their interactions included in the model.

If the interaction terms in the models were not statistically significant (*P* > 0.05), they were subsequently excluded from the final models. Differences were determined by *F*-tests using type III sums of squares. The PDIFF option and the Tukey test were applied as appropriate to evaluate pair-wise comparisons between the group means. Animal was the experimental unit and was included in the models as a random effect.

Any calves removed from the study were excluded from all data analysis.

## Results

### Clinical data, anthelmintic usage, animal performance in the FGS

Six calves (three control and three TST) were removed from the study during the FGS due to the following reasons; 1) two calves were removed due to lameness, 2) one calf died due to acute peritonitis, 3) one calf had diarrhoea and was ill-thrifty, 4) the dam of one calf was identified as a tuberculosis reactor using the intradermal skin test and was removed from the herd, and 5) the dam of one calf developed severe lameness so could not effectively nourish the calf.

With the exception of the pre-housing treatment on day 124, no calves in the TST groups were treated at pasture during the FGS.

**Fig. 1:**
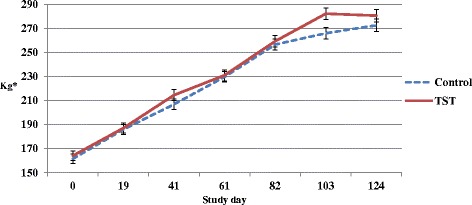
Live weight changes of suckler calves during the first grazing season

Calf ADG from birth to weaning for heifer and bull groups was 1.03 ± 0.02 and 1.08 ± 0.02 kg day^−1^, respectively (*P* = 0.03). Overall calf performance was similar between treatment groups (Fig. 1) as ADG ± s.e.m. for control and TST group calves during the FGS was 0.89 ± 0.02 and 0.94 ± 0.02 kg day^−1^, respectively (*P* = 0.17).

The ADG for control and TST group heifer calves during the FGS was 0.92 ± 0.03 and 0.89 ± 0.03 kg day^−1^, respectively (*P* = 0.94). The ADG for control and TST group bull calves during the FGS was 0.87 ± 0.03 and 0.98 ± 0.03 kg day^−1^, respectively (*P* = 0.06).

The ADG of calves born to Limousin × Friesian, Charolais × Limousin, Charolais × Simmental and Limousin × Simmental dams was 0.99 ± 0.03, 0.91 ± 0.03, 0.91 ± 0.03 and 0.86 ± 0.03 kg day^−1^, respectively, with significant differences in performance between calves born to Limousin × Friesian and Limousin × Simmental dams (*P* = 0.03).

### Clinical data, anthelmintic usage, animal performance in the SGS

At sampling on day 426, diarrhoea was observed in a number of heifers (*n* = 18) in one of the replicate groups. No clear clinical distinction could be made between TST and control heifers, with both appearing to be equally affected. Faecal samples from 12 diarrhoeic heifers (6 control and 6 TST) were analysed for the presence of fluke (liver and rumen) eggs. Eight heifers had both liver and rumen fluke eggs detected in their faeces with two additional heifers having rumen fluke eggs alone detected in their faeces. Based on both the clinical signs of diarrhoea and the laboratory results, all heifers (*n* = 46) were treated orally with oxyclozanide on day 433 (3 ml per 10 kg bodyweight, Zanil Fluke Drench, MSD Animal Health Ireland).

Two TST heifers had reached the treatment threshold on day 426 (PP ≥ 2.0 Utyr and FEC ≥ 200 epg) and as a result were treated subcutaneously with ivermectin on day 431.

Two other TST heifers exhibited clinical signs of respiratory disease (coughing and tachypnoea) on day 428. Both heifers were clinically examined and individual rectal faecal samples were collected and subsequently examined for the presence of lungworm larvae. Both were treated on day 428 with ivermectin and amoxicillin (1.0 ml per 10 kg bodyweight, Betamox LA 150 mg/ml Suspension for Injection, Norbrook Laboratories Limited). One of the two heifers was subsequently confirmed as having a patent lungworm infection. On day 431, coughing was widespread in both heifer groups but particularly in TST heifers. At this stage, all heifers (both control and TST), apart from the two treated on day 428, were rectal faecal sampled and treated with ivermectin. Faecal samples were examined for the presence of lungworm larvae using the modified Baermann technique. All faecal samples were negative for the presence of lungworm larvae.

**Fig. 2:**
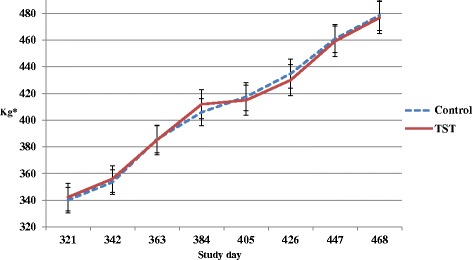
Live weight changes of yearling beef heifers during the second grazing season

Live weight ± s.e.m. of control and TST heifers at turnout on day 321 was 340.2 ± 9.54 and 342.3 ± 10.23 kg, respectively (*P* = 0.89) (Fig. 2). The ADG for control and TST heifers from turnout on day 321 to anthelmintic treatment on day 431 was 0.90 ± 0.04 and 0.80 ± 0.04 kg day^−1^, respectively (*P* = 0.03). The ADG for control and TST heifers from turnout on day 321 to housing on day 468 was 0.94 ± 0.03 and 0.90 ± 0.03 kg day^−1^, respectively (*P* = 0.34).

### Copper status

The mean ± s.e.m. calf plasma copper concentrations on days 0 and 124 were 13.30 ± 0.62 and 3.4 ± 0.29 μmol/l, respectively.

The mean yearling heifer plasma copper concentrations on days 251, 342 and 384 were 20.95 ± 0.54, 14.82 ± 0.36 and 11.93 ± 0.49 μmol/l, respectively.

There were no significant differences between treatments for plasma copper concentrations during either the FGS (*P* = 0.09) or the SGS (*P* = 0.19).

### Parasite status

In the FGS, there was an effect of time (*P* = 0.05), treatment (*P* < 0.01) and a treatment × time interaction (*P* = 0.04) for FEC (Table 1). There was an effect of time (*P* < 0.01), a treatment × time interaction (*P* < 0.01) and no effect of treatment (*P* = 0.53) for PP. Both FEC and PP were greater in TST calves than in control calves during the FGS.

**Table 1:** Faecal egg counts (FEC) and plasma pepsinogen (PP) concentrations in positive control and targeted selective treatment (TST) suckler beef calves in their first grazing season

		Date and day (d) of study							*P*-values			
Variable	Treatment	28-Jun-12	17-Jul-12	8-Aug-12	28-Aug-12	18-Sep-12	9-Oct-12	30-Oct-12	TRT	Time	TRT × Time	TRT × Time × Sex
	(TRT)	d 0	d 19	d 41	d 61	d 82	d 103	d 124				
FEC (epg)	Control	73^a,x^ (24.4)	51^a,x^ (24.3)	66^a,x^ (24.3)	64^a,x^ (24.3)	209^a,x^ (24.3)	95^a,x^ (24.3)	64^a,x^ (24.3)	< 0.01	0.05	0.04	0.61
	TST	57^a,x^ (24.3)	102^a,y^ (24.3)	75^a,x^ (24.3)	156^a,y^ (24.3)	415^b,x^ (24.3)	258^a,y^ (24.3)	172^a,x^ (24.4)				
PP (Utyr)	Control	0.40^a,x^ (0.03)	0.23^b,x^ (0.03)	0.29^a,x^ (0.03)	0.29^a,x^ (0.03)	0.30^a,x^ (0.03)	0.39^a,x^ (0.03)	0.28^a,x^ (0.03)	0.53	< 0.01	< 0.01	0.50
	TST	0.43^a,x^ (0.03)	0.25^b,x^ (0.03)	0.14^b,y^ (0.03)	0.37^a,x^ (0.03)	0.45^a,x^ (0.03)	0.45^a,x^ (0.03)	0.27^b,x^ (0.03)				

The values are expressed as Lsmeans (± s.e.m.). ^a,b,^ means within row and within measured variable not having a common superscript differ (*P* < 0.05) from day 0. ^x,y,^means within columns and within measured variable not having a common superscript differ (*P* < 0.05). Eggs per gram of faeces = epg. International units of tyrosine/litre = Utyr. Control calves were treated subcutaneously with ivermectin on days 0, 41 and 82. Targeted selective treatment calves were treated with the same product when one of the following thresholds were met; 1), positive for lungworm larvae using the modified Baermann technique or 2), positive or negative for lungworm larvae using the modified Baermann technique with PP ≥ 2 Utyr and FEC ≥ 200 epg

In the SGS, FEC was affected by both treatment (*P* = 0.07) and time (*P* < 0.01) but no treatment × time interaction occurred (*P* = 0.11) (Table 2). Plasma pepsinogen concentrations changed significantly over time (*P* < 0.01) and by treatment (*P* = 0.01), but no treatment × time interaction occurred (*P* = 0.94). Similar to the FGS, both FEC and PP were greater in TST animals than in control animals during the SGS.

**Table 2:** Faecal egg counts (FEC) and plasma pepsinogen (PP) concentrations in positive control and targeted selective treatment (TST) suckler beef heifers in their second grazing season

		Date and day (d) of study						*P*-values		
Variable	Treatment	15-May-13	5-Jun-13	26-Jun-13	17-Jul-13	7-Aug-13	28-Aug-13	TRT	Time	TRT × Time
	(TRT)	d 321	d 342	d 363	d 384	d 405	d 426			
FEC (epg)	Control	126^a,x^ (27.0)	54^a,x^ (19.8)	21^b,x^ (25.4)	50^a,x^ (25.3)	13^b,x^ (20.2)	13^b,x^ (13.6)	0.07	< 0.01	0.11
	TST	170^a,x^ (29.0)	70^b,x^ (21.3)	98^a,x^ (27.3)	83^a,x^ (27.2)	40^b,x^ (21.7)	45^b,x^ (14.6)			
PP (Utyr)	Control	0.62^a,x^ (0.04)	0.64^a,x^ (0.05)	1.11^b,x^ (0.07)	0.98^a,x^ (0.10)	1.20^b,x^ (0.13)	1.90^b,x^ (0.17)	0.01	< 0.01	0.94
	TST	0.71^a,x^ (0.04)	0.72^a,x^ (0.05)	1.27^b,x^ (0.07)	0.96^a,x^ (0.10)	1.50^b,x^ (0.14)	2.19^b,x^ (0.18)			

The values are expressed as Lsmeans (± s.e.m.). ^a,b,^means within row and within measured variable not having a common superscript differ (*P* < 0.05) from day 321. ^x,y,^means within columns and within measured variable not having a common superscript differ (*P* < 0.05). Eggs per gram of faeces = epg. International units of tyrosine/litre = Utyr. Control heifers were treated subcutaneously with ivermectin on day 393. Targeted selective treatment heifers were treated with the same product if PP ≥ 2.0 Utyr and FEC was ≥ 200 epg

### Pasture burdens and faecal cultures

Mean pasture larval counts on days 319, 341, 361, 382, 404, 424 and 445 were 159, 66, 296, 417, 57, 69 and 579 L_3_ /kg DM, respectively. Individual paddock burdens in the SGS ranged from 29 to 973 L_3_ /kg DM.

In the SGS, *Cooperia and Ostertagia* were the two main genera recovered in heifer faecal cultures on each sampling occasion (Table 3). It was observed that while *Cooperia* was the predominant nematode genus identified in cultures from faecal samples taken on day 405, *Ostertagia* was the main genus identified from faecal samples taken on day 426.

**Table 3:** Faecal larval culture results for heifers in their second grazing season

		Nematode genus		
		*Cooperia* spp*.*	*Ostertagia* spp*.*	*Trichostrongylus* spp*.*
Day (d) of study		% contribution of each genus		
d 384	Control	72	28	0
	TST	27	73	0
d 405	Control	100	0	0
	TST	77	23	0
d 426	Control	5	90	5
	TST	38	50	12

Targeted selective treatment = TST

### Lungworm ELISA

Using an optical density ratio (ODR) ≥ 0.5 indicating patent lungworm infection, three calves (two control and one TST) were seropositive on day 0. This contrasted with 28 out of the 93 calves (18 TST and ten control) being identified as seropositive on day 124. At sampling on day 468 at the end of the SGS, four heifers were serologically positive (three TST and one control).

## Discussion

To the authors’ knowledge, this is the first study to examine the use of a TST approach to nematode control in suckler beef cattle conducted over both their FGS and SGS. Excluding the pre-housing anthelmintic treatment which was common to both treatment groups in the FGS, no TST calves were treated at pasture in the FGS and yet achieved similar levels of performance to control calves. In the SGS, the TST approach had to be discontinued on day 431 when all heifers were treated with anthelmintics due to clinical signs of respiratory disease in both treatment groups, but particularly in TST heifers. Up to this point, only two TST heifers had reached treatment thresholds (two heifers had PP ≥ 2.0 Utyr and FEC ≥ 200 epg on day 426).

Previous studies examining TST-based approaches for the control of nematode infections in FGS beef cattle have been conducted in dairy-bred beef cattle using live weight gain as a TST measure [15, 16]. However, in the absence of any previous investigations on a TST-based approach to nematode control in suckler beef cattle over two grazing seasons, parasite-based indicators of infection were used in our study. Therefore, a combination of PP, FEC and the presence/absence of lungworm larvae in faecal samples were used in the FGS as TST treatment thresholds. As heifers were vaccinated in the SGS to prevent dictyocaulosis from occurring, PP and FEC were used in combination as TST treatment thresholds [3].

*Cooperia oncophora,* which is the predominate *Cooperia* species under these temperate conditions, was not considered in our treatment approach as given its mild pathogenicity it was expected to have relatively minor influence on animal performance. Although under conditions of heavy *Cooperia oncophora* challenge animal performance may be somewhat impaired, such a situation is unlikely to occur given the epidemiology of PGE in suckler beef systems. Furthermore, as it is an intestinal-dwelling nematode only FEC and not PP would be expected to increase as a result of challenge.

In a previous study [3], we reported that live weight gain may not be suitable as a TST measure for use in FGS suckler beef calves due to minimal differences in live weight gain between calves despite significant differences in FEC. The results of the present study are in agreement with these previous findings as FEC was significantly greater in TST calves compared to control calves whereas ADG was similar across treatments. Calf ADG from birth to weaning for heifer and bull calves (1.03 and 1.08 kg day^−1^, respectively) was similar to other Irish studies performed on rotationally grazed spring-calving suckler beef herds [24, 25]. As dam milk yield is a key determinant of calf performance [26], the finding that ADG from birth to weaning was greater in calves born to Limousin × Friesian dams was not surprising given their greater milk yield compared to other suckler cow breed types [27]. It is however noteworthy that overall ADG from birth to weaning in the present study was comparable to other studies given the incidence of hypocupraemia in calves on day 124 [28] as such a deficiency of copper can be associated with impaired performance [29]. It has previously been reported that copper deficiency may only result in reduced calf ADG in the presence of molybdenum [30] and although the level of molybdenum in soil or grass samples was not measured, it may be a possible explanation for the lack of effect of hypocupraemia on calf performance in the present study.

In the SGS, heifers were vaccinated before turnout on day 321 to prevent dictyocaulosis from occurring. This lungworm vaccine, which has been commercially available for approximately 50 years [31], reduces both faecal larval counts and the risk of mortality due to dictyocaulosis [32]. However, the immunity that develops after vaccination is not complete [31] and this was evidenced by the detection of patent lungworm infection in one heifer during the SGS.

Although FGS calves can be successfully vaccinated at pasture to prevent dictyocaulosis from occurring if performed early in the grazing season [32, 33], calves in the present study were not vaccinated during the FGS given the time of year they were born. As the youngest calf in the study was born on day −58, an effective immunity would not have been established by the time when challenge with lungworm would most likely have occurred [34]. Based on the results of the lungworm ELISA conducted at both the start of the study (day 0) and again at day 124, 25 calves seroconverted during the FGS. This level of seroconversion was surprising given that no calves had lungworm larvae detected in their faeces using the modified Baermann technique in the FGS. This highlights that lungworm may circulate at low levels of infection in suckler beef calves during the FGS.

The clinical signs of respiratory disease (coughing, tachypnoea) observed in heifers during the SGS, which abated within four to five days of treatment with ivermectin, resembled the reinfection syndrome [20]. This is evidenced by the fact that only one heifer had lungworm larvae detected in faecal samples at the time clinical signs were observed while four heifers were identified as being seropositive based on the results of the lungworm ELISA conducted on blood samples taken from heifers at housing on day 468. As control heifers were treated with ivermectin on day 393 and considering that ivermectin prevents reinfection with lungworm for a minimum period of three weeks [35], this may help to explain the apparent higher incidence of respiratory disease in TST heifers in contrast to control heifers.

It is difficult to establish in the present study the relative importance of the detection of both rumen and liver fluke egg in faecal samples and the presence of diarrhoea in heifers on day 426. A more likely explanation is that diarrhoea was due to challenge with *Ostertagia* as this was the main nematode identified in faecal cultures of both control and TST heifers sampled on day 426 while mean PP on day 426 were 1.9 and 2.2 Utyr in control and TST groups, respectively. A previous study [36], using the same method for PP determination as the present study found that when mean PP in a group of calves at pasture exceeded 2.0 Utyr, calves began exhibiting clinical signs of PGE. In a study using suckler beef cattle in their SGS, PGE was observed when mean PP were greater than 2.0 Utyr [8]. Similar to that study [8], diarrhoea was evident in animals in the present study despite FEC being relatively low. This can be explained by the poor fecundity of *Ostertagia* spp. [37].

Pasture larval burdens in the present study never exceeded 1000 L_3_ /kg DM. Although clinical signs of PGE are only expected to occur above a threshold of 5000 L_3_ /kg DM [38], the presence of clinical disease in the present study may be explained by the fact that the same paddocks were not sampled every three weeks. Paddocks that were sampled at the three week intervals were identified on the basis of their pre-grazing sward height approximately three days before heifer groups were moved into them. It is therefore possible that paddocks with high burdens may have been missed during the sampling process.

It has previously been observed that unlike autumn- or winter-born suckler beef calves, spring- or summer-born suckler beef calves may experience minimal GIN exposure in their FGS as calves only start consuming appreciable quantities of grass when pasture burdens of overwintered larvae, which developed from nematode eggs deposited during the previous grazing season, would have declined [4]. Previously, it was hypothesised that a mean PP in FGS calves at housing less than a range of 1.5 to 2.0 Utyr was suggestive of insufficient exposure to GIN challenge in the FGS [39]. On day 124, mean PP in both control and TST calves were 0.3 Utyr. These PP values are similar to those recorded in parasite-naive calves [40] and this lack of GIN exposure in the FGS negatively interfered with their immune development.

In the SGS, there was a significant difference in ADG between control and TST heifers from turnout on day 321 to anthelmintic treatment on day 431 (0.90 and 0.80 kg day^−1^, respectively). In contrast, ADG from turnout to housing in the SGS for both groups was similar. It would appear that the anthelmintic treatment given to heifers on day 321 benefitted the TST heifers in particular, as judged by the similar levels of performance of control and TST heifers when performance was measured over the whole grazing season.

As with all pasture-based studies, the research findings presented here need to be interpreted with caution given that many local factors can ultimately influence the parasite-host interaction. Examples of these local factors include climate, stocking rates, turnout dates and previous pasture history.

Due to unfavourable weather conditions in the SGS, heifers were turned out to pasture approximately ten weeks later than the usual turnout date. This delayed turnout would undoubtedly have affected the build-up of infective larvae on pasture. We can only surmise that pasture larval burdens would have been considerably greater if heifers had been turned out to pasture as per normal. However, it would be difficult to predict the extent to which this increased pasture larval challenge would have had on either heifer performance or on the incidence of clinical parasitic disease within the two grazing groups. Thus, there is a clear need to conduct further research to determine the magnitude of these potential effects.

## Conclusion

Parasite control in FGS suckler beef calves at pasture can potentially be controlled with fewer anthelmintic treatments without negatively impacting performance. The results of the SGS study highlight that many factors can ultimately affect the performance of cattle at pasture. This was evidenced by challenge due to lungworm, rumen fluke and challenge due to abomasal nematodes. Based on the results of the present study, it would appear that spring-born suckler beef calves require a minimum of one anthelmintic treatment in their SGS to maintain performance. This is a result of the minimal exposure to nematode challenge in the FGS which makes them more susceptible to parasitic disease in their SGS.
